# Application of Response Surface Methodology for Optimizing the Therapeutic Activity of ZnO Nanoparticles Biosynthesized from *Aspergillus niger*

**DOI:** 10.3390/biomimetics6020034

**Published:** 2021-05-27

**Authors:** Ali Es-haghi, Mohammad Ehsan Taghavizadeh Yazdi, Mohammad Sharifalhoseini, Mohsen Baghani, Ehsan Yousefi, Abbas Rahdar, Francesco Baino

**Affiliations:** 1 Department of Biology, Mashhad Branch, Islamic Azad University, Mashhad 9187147578, Iran; baghanigraphic@yahoo.com (M.B.); yousefi.esn@gmail.com (E.Y.); 2 Applied Biomedical Research Center, School of Medicine, Mashhad University of Medical Sciences, Mashhad 9177948564, Iran; metyazdi@gmail.com; 3 Department of Biology, Damghan Branch, Islamic Azad University, Damghan 3671637849, Iran; mohammadsharifalhoseini@yahoo.com; 4 Department of Physics, School of Basic Sciences, University of Zabol, Zabol 9861335856, Iran; 5 Department of Applied Science and Technology, Institute of Materials Physics and Engineering, Politecnico di Torino, 10129 Torino, Italy

**Keywords:** zinc oxide, nanomaterials, response surface methodology, anticancer, antimicrobial, optimization

## Abstract

In this study, the biosynthesis of zinc oxide nanoparticles using *Aspergillus niger* (A/ZnO-NPs) is described. These particles have been characterized by UV–Vis spectrum analysis, X-ray powder diffraction, field emission scanning electron microscopy, and transmission electron microscopy. To use this biosynthesized nanoparticle as an antiproliferative and antimicrobial agent, the IC_50_ value against the breast cancer cell line and inhibition zone against *Escherichia coli* were used to optimize the effect of two processing factors including dose of filtrate fungi cell and temperature. The biosynthesized A/ZnO-NPs had an absorbance band at 320 nm and spherical shapes. The mean particles size was 35 nm. RSM (response surface methodology) was utilized to investigate the outcome responses. The Model F-value of 12.21 and 7.29 implies that the model was significant for both responses. The contour plot against inhibition zone for temperature and dose showed that if the dose increases from 3.8 to 17.2 µg/mL, the inhibition zone increases up to 35 mm. As an alternative to chemical and/or physical methods, biosynthesizing zinc oxide NPs through fungi extracts can serve as a more facile and eco-friendly strategy. Additionally, for optimization of the processes, the outcome responses in the biomedical available test can be used in the synthesis of ZnO-NPs that are utilized for large-scale production in various medical applications.

## 1. Introduction

Nano-science and nano-technology rely on the understanding, manipulation, and usage of matter at the nanoscale and could be potentially used in all of the scientific disciplines including biochemistry, biomaterials, and bioengineering [1,2,3,4,5]. Nanoparticles have unique attributes and exclusive structure [6,7,8,9,10]. A number of nanoparticles (NPs) including metal-oxide nanoparticles could be applied in biomedical applications [11,12,13,14]. Zinc-oxide NPs (ZnO-NPs) are among the most important in various research fields [15]. ZnO is an extensive band-gap semi-conductor, which has been used in several areas [3]. ZnO-NPs have emerged as a promising possibility in biomedical sciences, particularly in anti-cancer and anti-microbial fields, where their ability to trigger extra-ROS (reactive oxygen species) production and release zinc ions may play a role in cell apoptosis [16,17]. ZnO-NPs have low or non-toxicity. Nevertheless, higher levels of ZnO-NPs show greater cell toxicity in cancerous cell lines. Mechanisms of cell toxicity of ZnO-NPs cause zinc-mediated protein activity disequilibrium and oxidative stress, finally killing the cell [18]. Results have revealed that ZnO-NPs stimulate cytotoxicity in cancer cells (WEHI-3) [19], neural stem cells in mouse and human [20,21], osteoblast cancer cells [22], and tumor cell lines (HeLa, A549, SK-MES-1, and NCI-H460) [23]. ZnO-NPs can be synthesized using chemical and biological methods [24]. Generally, biological synthesis of metal-oxide NPs is a more proper solution than chemical methods of synthesis as it exhibits several advantages like using low-cost, non-toxic chemicals [25,26,27]. Bio-agents are usually ecofriendly and can act as reducing agents of metal ions, thus being applied for the synthesis of small and stable ZnO-NPs [28]. Microorganisms like yeast, bacteria, algae, and fungi play an important role in the reducing metals either intra- or extracellularly, which forms the basis for the use of microorganisms in the biosynthesis of nanoparticles using eco-friendly methods and act as interesting nano-factories [29]. These strategies are used for the synthesis of different metal nanoparticles like silver, gold, and zinc [30,31]. Attention has been paid to fungi as candidates for NP synthesis. Fungi have advantages over other microorganisms for the biosynthesis of nanoparticles due to high tolerance toward metals and high wall binding capacity as well as intracellular metal uptake capabilities; their high secretion of proteins, enzymes, and metabolites; high growth rates; easy handling in large-scale production; and low-cost requirements for production procedures. In addition, nanoparticles synthesized using fungi present fair mono-dispersity and stability compared to other microorganisms [32]. The exact mechanism for the production of NPs is still partly unclear, but the existence of bio-molecules in the biomass may be used for the production of NPs. For example, fungi release huge quantities of enzymes and are easy-growing, thus they are regarded as a suitable source for the synthesis of NPs. Bio-modeling and optimization can help to better understand and perform biological tests [33,34,35]. In the present study, the optimization of ZnO-NP synthesis using IC_50_ values against breast cancer cells and *Escherichia coli* bacteria for two characteristics including temperature and dose of filtered fungal cells is demonstrated.

## 2. Experimental

### 2.1. Materials

A registered *Aspergillus niger* strain (PTCC 5012) was obtained from the Persian Type Culture Collection (PTCC, Tehran, Iran). The fungi were inoculated in solution media containing: glucose (10 g/L), KH_2_HPO_4_ (7.0 g/L); K_2_HPO_4_ (2 g/L); MgSO_4_·7H_2_O (0.1 g/L); (NH4)_2_SO_4_ (1 g/L); yeast extracts (0.6 g/L). The fungus was placed inside the flask and an orbital shaker was used to incubate the fungus at 150 rpm at the temperature of 37 °C for 96 h. The culture of the fungi was then filtered through Whatman no. 1 paper. The cell filtrate was used for NP synthesis. 

### 2.2. Green Synthesis and Characterization of A/ZnO-NPs

Zinc acetate dihydrate (Zn(CH_3_COO)_2_·2H_2_O of analytical grade was used as a source to synthesize ZnO-NPs. For the synthesis of NPs, 50 mL of zinc acetate dihydrate solution (1 mM) was mixed and stirred with different doses of cell filtrate and agitated at different temperatures based on the experimental design in the dark. After that, the gained solution was centrifuged at 10,000 rpm for 5 min. The precipitate was gathered, washed once with 96% ethanol, twice with deionized water, and then dried for characterization. 

Characterization of A/ZnO-NPs was performed by using several techniques such as UV–Visible spectrophotometry (UVD-3200, LABOMED, USA), field-emission scanning electron microscopy (JEOL, Tokyo, Japan), transmission electron microscopy (Leo 912 AB Zeiss, Oberkochen, Germany), and powder X-ray diffraction (D8 ADVANCE-BRUKER, Billerica, MA, USA).

### 2.3. Cytotoxicity Effects

MCF7 and skin fibroblast cells were treated with 20 µg/mL of A/ZnO-NPs. The nanoparticles were suspended in deionized water. After the incubations, the MTT test was done and absorbance was measured at 570 nm with plate-reader spectroscopy (Epoch, Biotek, Winooski, VT, USA). The cell-viability percentage was measured by this ratio: (A treated/A control) × 100, where A is the absorbance of the cells.

### 2.4. Antimicrobial Activity

The antimicrobial activity of A/ZnO-NPs was evaluated against *Escherichia coli* (MTCC No. 739) by the agar diffusion test [36]. Mueller–Hinton agar plates were cultured with 100 μL of grown broth cultures of the respective test bacteria. Sterile readymade discs were loaded with 20 µg/mL A/ZnO-NPs separately. The nanoparticles were suspended in deionized water. The plates were incubated for 48 h. The growth of the inhibition zone around the extract-loaded discs was recorded in millimeters (diameter of the zone). 

### 2.5. Experimental Plan

In order to control the optimum status for biosynthesis of ZnO-NPs, dose of filtrate fungi cell and temperature as the impelling agents were selected and the central composite design (CCD) was applied. This design allowed us to establish the optimal rate of the substantial factors and the interactions of such variables in the process. A three-level CCD with four replicates at the center point with 13 runs was applied. Tested variables (IC_50_ and inhibition zone) were denoted as x1 and x2, respectively. The principle of response surface methodology (RSM) has been explained [37] with the objective of optimization of the response based on the factors evaluated. The arrangement of CCD permits the expansion of an empirical second-order polynomial multiple regression models. In order to specify the significance of the model, ANOVA (analysis of variance) was conducted. The response surface and contour plots of the model-predicted responses were applied to specify the interactive relations between the significant variables. Design Expert, v. 8.0.7.1 (Stat-Ease Inc., Minneapolis, MN, USA) was used to design the tests and for regression and graphical analysis of the obtained data.

## 3. Results and Discussion

**Figure 1:**
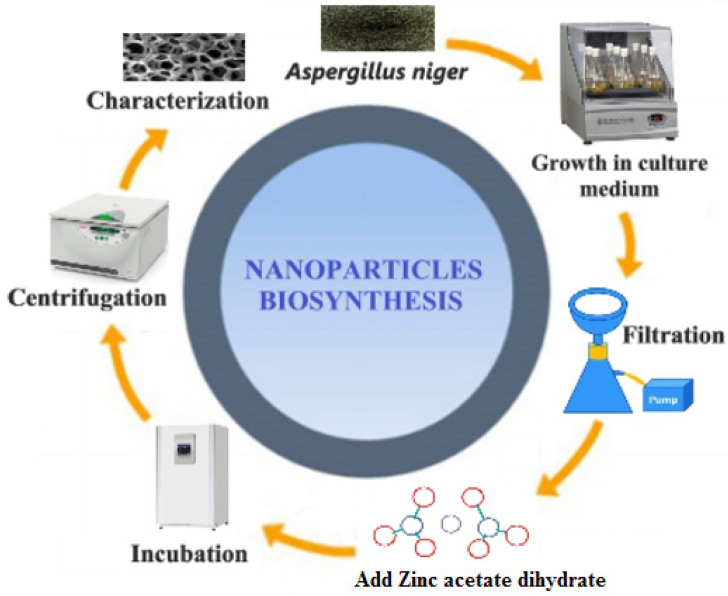
A tentative mechanism for the formation of ZnO-NPs by *A. niger*.

Green production of metal-oxide NPs utilizing biomass constituents is appealing because these methods are uncomplicated, cheap, and non-toxic compared to chemico-physical procedures [38,39,40,41]. The current study reports the extracellular synthesis of A/ZnO-NPs via fungi. The biochemical procedure of NP shaping and stabilization has remained largely undiscovered, except for some investigation groups that have shown that the proteins observed in enzymes released by microorganisms are the chief biomolecules included in the formation of metal/metal oxide NPs. Figure 1 summarizes the mechanism of synthesis of ZnO-NPs by *A. niger*.

### 3.1. Characterization of A/ZnO-NPs

**Figure 2:**
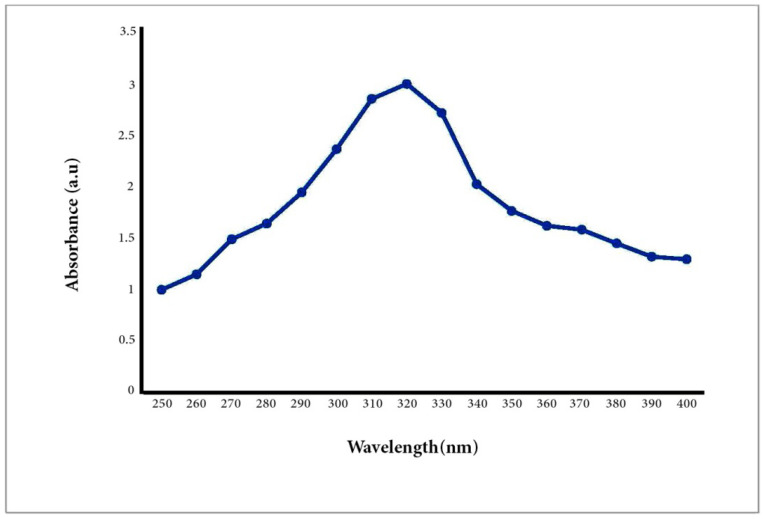
UV–Vis spectroscopy of the biosynthesized A/ZnO-NPs.

UV–Vis spectrum analysis is usually used to study the size and shape of NPs [42]. The rate and width of the surface plasmon absorbent rely on the size/shape of the NPs as well as on the dielectric constant of the metal itself and the surrounding medium. It is also well known that solutions containing ZnO-NPs exhibit a characteristic absorption peak below 400 nm. Therefore, the A/ZnO-NPs absorbance peak was identified using UV–Vis spectroscopy in the range of 250 to 400 nm. The UV–Vis spectrum analysis of the biosynthesized A/ZnO-NP specimen is displayed in Figure 2. It was revealed that the UV–Vis absorption spectra of green synthesized ZnO-NPs showed an absorption peak at 320 nm. The bandgap energy was calculated using the formula E_g_ = 1240/λ eV and was found to be 3.8 eV, which is comparable to the previously reported values of energy bandgap for ZnO nanoparticles [43,44]. It is worth noting that the obtained E_g_ value was different from the bandgap of bulk ZnO (3.37 eV); this result can be attributed to the optical confinement effect corresponding to the size and length of NPs [45].

**Figure 3:**
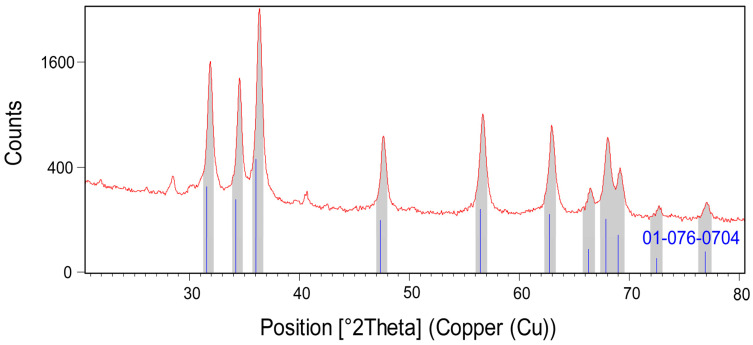
PXRD pattern of green synthesized A/ZnO-NPs.

Powder X-ray diffraction (PXRD) was conducted to examine the crystal structure of the optimized sample and crystallite size (Figure 3). The crystal structure was compatible with the reference pattern of zinc oxide with code JCPDS #01-076-0704. Hence, the corresponding (hkl) values were (100), (002), (101), (102), (110), (103), (200), (112), (201), (004), and (202). The calculated 20 values and intensities (%) were 31.74 (57), 34.38 (41.5), 36.22 (100), 47.48 (21.2), 56.54 (30.8), 62.78 (26.5), 66.30 (4.1), 67.87 (22), 69.01 (10.9), 72.47 (1.6), and 76.87 (3.4). The 2θ values of the synthesized A/ZnO-NPs were in close relation to the reference pattern, which had values of 31.90, 34.55, 36.35, 47.58, 56.62, 62.92, 66.44, 68.00, 69.18, 72.73, and 77.08°. The A/ZnO-NPs had a hexagonal crystal system and P63mc space group. The crystallite size of the nanoparticles was obtained by the Scherrer equation, which was calculated to be 35.51 nm.

**Figure 4:**
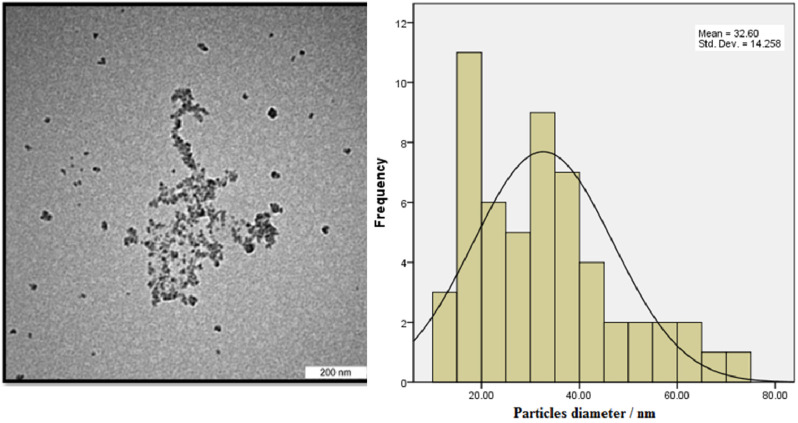
TEM image of the biosynthesized A/ZnO-NPs and its histogram of particle diameter.

**Figure 5:**
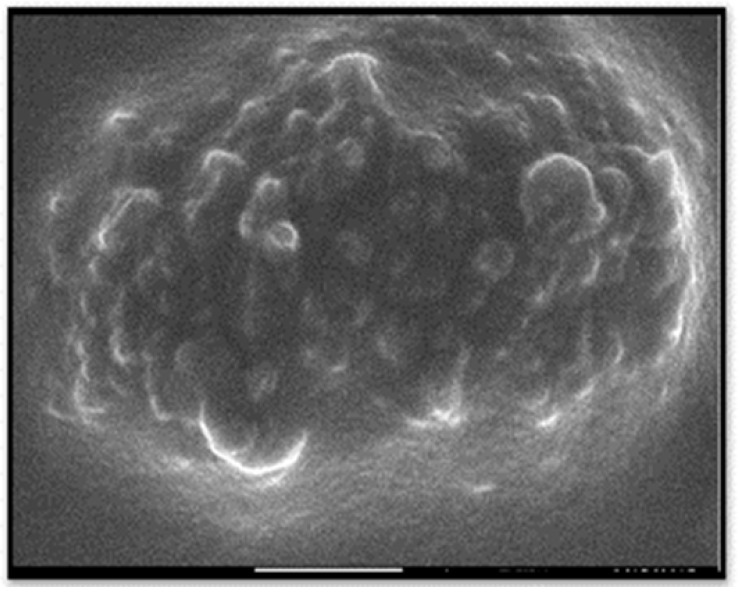
FESEM image of the biosynthesized A/ZnO-NPs.

The TEM image showed that the particles sizes ranged from 10 to 70 nm (Figure 4). The mean diameter of the biosynthesized nanoparticles measured by TEM was about 33 nm, which is in good accordance with the NP size estimated from the PXRD results. Dark shadows on the surface of the nanoparticles in the TEM image may be the bioorganic molecules/enzyme of fungi. The dimension, shape, and distribution of the as-synthesized A/ZnO-NPs were analyzed through FE-SEM monitoring. Figure 5 presents the FE-SEM image of A/ZnO-NPs, which were prepared after 24 h of incubation. A/ZnO-NPs showed a distorted spherical shape. This agglomeration is the caused by polarity and electrostatic attraction of ZnO-NPs. Regarding the fact that the specific properties of NPs depend on their shape, synthesis of NPs, along with controlling shape, is very important. In general, the most appropriate shape that can be used for biological purposes is the spherical shape, as the absence of sharp or cutting edges prevent damage to cells/tissues. 

Nanoparticles produced by plant and microorganism extracts are the center of attention because of their bio-compatibility and low cost [46,47]. ZnO displays significantly enhanced biomedical characteristics in its nano-forms. Working on the properties of ZnO-NPs revealed that their nanocomposites (NCs) had more potent biomedical and photocatalytic effects [48,49]. Bacteria were amongst the main organisms employed for the production of nanoparticles as a result of their feasibility of isolation and use [50]. Saravanan et al. generated anisotropic ZnO-NPs by *Bacillus megaterium* cell free extracts as unique reducing/capping agents [51]. The UV spectrum of the ZnO-NPs displayed the SPR peak at 346 nm, which is consistent with our results. In the other study, β-chitinous scaffolds were employed as a template for the formation of ZnO materials. The results showed the growth of ZnO nano-crystals on the β-chitin. The chitin/ZnO composites showed antibacterial properties against Gram-positive bacteria [52]. In another work, extracellular metabolites of *A. niger* were used to transform ZnO-NPs into a zinc oxalate [53]. The results show that due to the presence of high active metabolites, *A. niger* can biomineralize inorganic NPs and transform these NPs into more stable oxalate complexes. 

### 3.2. Central Composite Design

RSM is a statistical technique that uses quantitative data from proper tests to explain regression model equations. This is generally achieved by assessing which of the investigated variables and their interactions have more important effects. There are several variables that may affect the response of a system, and it is almost incredible to recognize and control them all. In this work, we studied how to optimize two responses induced by ZnO-NPs (i.e., the cytotoxic effects toward MCF-7 cells (IC_50_) and the anti-bacterial activity (inhibition zone) against *E. coli*). The effect of the two variables including the dose of filtrated fungi cell and temperature were studied. Two independent RSM models were developed to precisely structure the model and ascertain the impact of individual factors. The ANOVA was used to analyze the differences among means. Only those terms rendered significant results according to ANOVA were applied in the model. Central composite design (CCD) for the two variables and two experimental responses including IC_50_ and inhibition zone adapted after 13 runs is shown in Table 1.

ANOVA was done to obtain the best synthesis conditions for ZnO-NPs so that they had the highest cytotoxicity effects against cancer cells. The results are presented in Table 2. According to the data from Table 2, the quadratic model is the best model for both dose and temperature for the IC_50_ response. As shown in Table 2, the two factors, temperature (A) and dose (B), and combination of them was not significant (*p*-value > 0.05), but A^2^ and B^2^ were significant. F value of the quadratic model and individual model terms also helped in finding their significance. The F-value of the model of 12.21 showed that the model was significant (Table 2). *p*-values less than 0.05 highlighted that the model terms were significant. 

**Figure 6:**
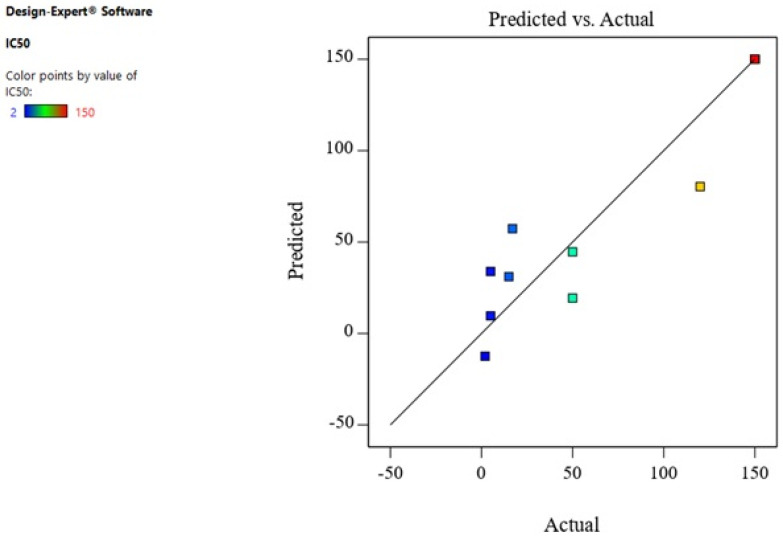
The predicted response values versus the actual response values for IC_50_.

Therefore, A² and B² were the significant model terms with F-values of 16.98 and 47.60 (Table 2). Hoseinpour also reported that extract ratio was a significant factor for the biosynthesis of ZnO-NPs from a Dittrichia aqueous extract [54]. Figure 6 displays the predicted response values in parallel to the actual response values. The purpose was to identify a value or group of values not easily predictable by the model. 

In order to obtain the effective factors to find the optimal conditions, linear regression was used. In linear regression, mean response and predicted response are values of the dependent variable measured from the regression parameters and a given value of the independent variable. The values of these two responses are similar, but their calculated variances are dissimilar. Table 3 shows the final equation regarding the actual factors.

Equations determine that the dose was the most influential factor with a positive result, and then temperature had positive effects on the IC_50_. Souri et al. indicated that the extract to metal ratio was the most effective parameter for the biosynthesis of manganese dioxide NPs by *Yucca gloriosa* leaf extract [55]. The normal probability plot is a graphical method for evaluating whether or not a dataset is approximately normally distributed. If the normal probability diagram depicts a straight line, the residuals have a normal distribution. The contour plot is a two-dimensional (2D) representation of the response plotted against combinations of numeric factors and/or mixture components. It can display the association between the responses, mixture components, and/or numeric factors.

**Figure 7:**
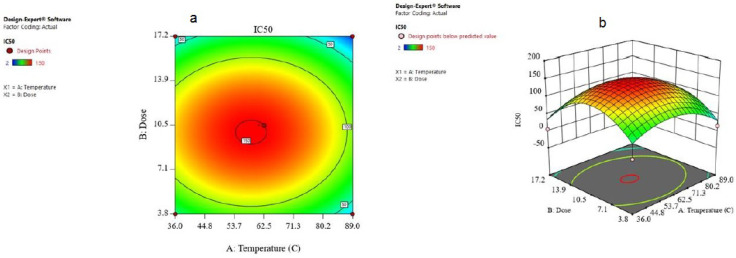
(**a**) The contour plot and (**b**) 3D surface plot for the dose and temperature for IC_50_ response.

**Figure 8:**
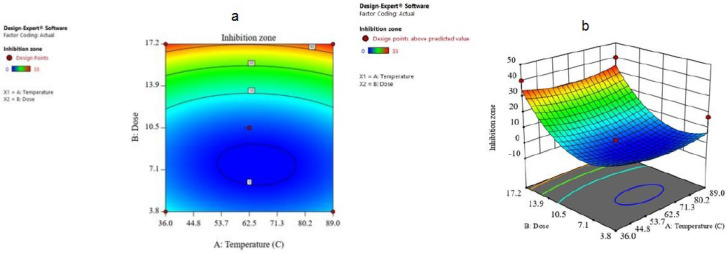
(**a**) The contour plot and (**b**) 3D surface plot for dose and temperature for inhibition zone.

The combined result of the dose and temperature was evaluated and the consequences were given in the form of contours and 3D plots (Figure 7a,b). The contour plot against IC_50_ for temperature and dose is shown in Figure 8a. Contour plots are a way to show a three-dimensional surface on a two-dimensional plane (Figure 7b). The 3D surface plot is a projection of the contour plot giving shape, in addition to the color and contour. Figure 8a,b shows that if the temperature increases from 36 to 89 °C and the dose increases from 3.8 to 17, the IC_50_ on the MCF-7 cells decreased from 150 to about 50, which means that it is in accordance with the model.

ANOVA analysis was also performed to obtain the best synthesis conditions for ZnO-NPs, leading to the greatest anti-bacterial effects. The results are presented in Table 4. According to the data from Table 4, the quadratic model is the best model for the inhibition zone response. As shown in Table 4, the term of dose (B) and B^2^ were significant (*p*-value ≤ 0.05), but the term of temperature (A) and A^2^ and the combination of them (AB) were not significant. The F-value of the model was 7.29, which reflects the significance of the model (Table 4). The probability of obtaining such a great F-value due to noise is only 1.07%. *p*-values < 0.05 revealed significant model terms including B and B² with the respective F-values of 1.40 and 19.20 (Table 4).

Final equation in terms of actual factors is shown in Table 5. The equation regarding the actual values can be utilized to anticipate the response for the levels of an individual factor.

The combined result of the dose and temperature was examined and the results are given in the form of contours and 3D plots (Figure 7a,b). The contour plot against inhibition zone for temperature and dose showed that if the dose increases from 3.8 to 17.2, the inhibition zone increases up to 35 mm, which means that it is in accordance with the model.

## 4. Conclusions

In this study, zinc oxide nanoparticles prepared using *Aspirgilous niger* extracts were optimized and estimated using design expert software. The ZnO-NPs were characterized via FESEM, TEM, and XRD analysis methods. Design of the response surface was selected to examine the main effects of the factors and their interactions. In this research, two variables factors, dose of filtrate fungi cell and temperature, were used to optimize the synthesis of ZnO-NPs for two responses, cytotoxicity against MCF-7 cell (IC_50_) and antibacterial activity (inhibition zone). The F-value of model was 12.21 and 7.29 for the IC_50_ value and inhibition zone, respectively, which implies that the model is significant. In the case of IC_50_, dose (B) and temperature (A) as well as combination of them were not significant (*p*-value > 0.05), but the A^2^ and B^2^ were significant. In the case of inhibition zone, the terms dose (B) and B^2^ were significant (*p*-value ≤ 0.05), but the terms of temperature (A) and A^2^ and the combination of them (AB) were not significant. The biosynthesized A/ZnO-NPs had an absorbance band at 320 nm and spherical shapes. The mean particle size was 35 nm. The contour plot against inhibition zone for temperature and dose showed that if the dose increased from 3.8 to 17.2 µg/mL, the inhibition zone increased up to 35 mm. As an alternative to physico-chemical methods, producing ZnO-NPs through fungi can serve as a more simplistic and eco-friendly plan. Moreover, for optimization of the processes, the outcome responses in the biomedically available test can be used in the synthesis of ZnO-NPs that are utilized for large-scale production in various medical applications.

## Figures and Tables

**Table 1:** Central composite design (CCD) for the two variables (temperature and dose of biosynthesized NPs) and two experimental responses (IC_50_ and inhibition zone).

	Factor 1	Factor 2	Response 1	Response 2
Std	A: Temperature (°C)	B:Dose	IC_50_	Inhibition zone (mm)
1	36.0	3.8	17	20
2	89.0	3.8	15	17
3	36.0	17.2	5	40
4	89.0	17.2	5	40
5	25.0	10.5	120	2
6	100.0	10.5	50	2
7	63	1.0	50	2
8	63	20.0	2	46
9	63	10.5	150	2
10	63	10.5	150	2
11	63	10.5	150	2
12	63	10.5	150	2
13	63	10.5	150	2

**Table 2:** ANOVA of the response surface quadratic model for IC_50_.

Source	Sum of Squares	df	Mean Square	F-Value	*p*-Value
**Model**	47,886.85	5	9577.37	12.21	0.0024
**A-Temperature**	1275.00	1	1275.00	1.63	0.2430
**B-Dose**	1009.85	1	1009.85	1.29	0.2938
**AB**	1.00	1	1.00	0.0013	0.9725
**A²**	13,315.22	1	13,315.22	16.98	0.0045
**B²**	37,325.65	1	37,325.65	47.60	0.0002
**Residual**	5489.15	7	784.16		

**Table 3:** Final equation in terms of actual factors.

IC_50_	=
−222.86128	
+7.27221	Temperature
+32.24067	Dose
+0.002807	Temperature × Dose
−0.062222	(Temperature)²
−1.62327	(Dose)²

**Table 4:** ANOVA for response surface quadratic model for the inhibition zone.

Source	Sum of Squares	df	Mean Square	F-Value	*p*-Value
**Model**	2996.86	5	599.37	7.29	0.0107
**A-Temperature**	1.13	1	1.13	0.0137	0.9102
**B-Dose**	1384.05	1	1384.05	16.84	0.0046
**AB**	2.25	1	2.25	0.0274	0.8733
**A²**	114.81	1	114.81	1.40	0.2759
**B²**	1578.29	1	1578.29	19.20	0.0032
**Residual**	575.45	7	82.21		

**Table 5:** Final equation in terms of actual factors.

Inhibition Zone	=
+44.45795	
−0.780575	Temperature
−5.31481	Dose
+0.004211	Temperature × Dose
+0.005778	(Temperature)²
+0.333795	(Dose)²

## Data Availability

Data are included within this article.
